# Time with friends and physical activity as mechanisms linking obesity and television viewing among youth

**DOI:** 10.1186/1479-5868-12-S1-S6

**Published:** 2015-07-27

**Authors:** Elizabeth A Vandewater, Seoung Eun Park, Emily T Hébert, Hope M Cummings

**Affiliations:** 1 Michael & Susan Dell Center for Healthy Living, The University of Texas School of Public Health Austin Regional Campus, 1616 Guadalupe St., Austin, TX 78701, USA; 2 Harvard University, 677 Huntington Avenue, Kresge Building, 7th Floor, Boston, MA 02115, USA

**Keywords:** youth obesity, television viewing, time with friends, physical activity

## Abstract

**Background:**

Though bivariate relationships between childhood obesity, physical activity, friendships and television viewing are well documented, empirical assessment of the extent to which links between obesity and television may be mediated by these factors is scarce. This study examines the possibility that time with friends and physical activity are potential mechanisms linking overweight/obesity to television viewing in youth.

**Methods:**

Data were drawn from children ages 10-18 years old (M = 13.81, SD = 2.55) participating in the 2002 wave of Child Development Supplement (CDS) to the Panel Study of Income Dynamics (PSID) (n = 1,545). Data were collected both directly and via self-report from children and their parents. Path analysis was employed to examine a model whereby the relationships between youth overweight/obesity and television viewing were mediated by time spent with friends and moderate-to-vigorous physical activity (MVPA).

**Results:**

Overweight/obesity was directly related to less time spent with friends, but not to MVPA. Time spent with friends was directly and positively related to MVPA, and directly and negatively related to time spent watching television without friends. In turn, MVPA was directly and negatively related to watching television without friends. There were significant indirect effects of both overweight/obesity and time with friends on television viewing through MVPA, and of overweight/obesity on MVPA through time with friends. Net of any indirect effects, the direct effect of overweight/obesity on television viewing remained. The final model fit the data extremely well (χ^2^ = 5.77, df = 5, p<0.0001, RMSEA = 0.01, CFI = 0.99, TLI =0.99).

**Conclusions:**

We found good evidence that the positive relationships between time with friends and physical activity are important mediators of links between overweight/obesity and television viewing in youth. These findings highlight the importance of moving from examinations of bivariate relationships between weight status and television viewing to more nuanced explanatory models which attempt to identify and unpack the possible mechanisms linking them.

## Background

Despite recent reports suggesting that childhood obesity rates in the U.S. may be leveling off or starting to decline [1], obesity among children and youth continues to be one of the most persistent public health problems in the United States. Approximately 22.8% of 2- to 5-year-olds, 34.2% of 6- 11-year-olds, and 34.5% of 12-19 year-olds are overweight or obese [1]. Obesity in childhood is of particular concern because it is linked to both immediate and long-term health problems and tends to persist into adulthood [2,3]. Unless reversed, the current rates of obesity among U.S. children and youth will dramatically affect public health well into the middle of the 21^st^ century.

Over the past 35 years or so, an enormous amount of intellectual and financial capital has been spent on identifying and understanding the factors contributing to the obesity epidemic. Early on, television viewing was identified as a possible major contributor to childhood obesity. In 1985, Dietz and Gortmaker reported that the prevalence of obesity among adolescents aged 12-17 (drawn from the cross-sectional National Health and Nutrition Examination Surveys or NHANES) increased 2% with each hour of television viewed [4]. They proposed that television viewing plays a causal role in childhood obesity—a view that was largely accepted then and now. Thirty years later, this study is still widely cited as definitive evidence that television causes obesity in youth, and a voluminous body of research aimed at replicating this result has been produced.

Yet, empirical evidence for the link between television and obesity has been surprisingly inconsistent. Despite high levels of media use and a high incidence of obesity among youth, evidence that these concurrent trends are strongly related is mixed at best [5]. Some studies find weak but positive associations between television viewing and obesity among youth [6-9], some find no relationship [10-12], and others find that the relationship is no longer statistically significant once important confounders are controlled for [13-15]. In a meta-analysis, Marshall, Biddle, Gorely, Cameron & Murdey found that the associations between media use and obesity among youth, though consistently positive, tend to be extremely weak, and concluded that they are of little clinical relevance [16]. Though this debate is by no means settled, the notion that media use must somehow be responsible for the increased prevalence of obesity in American children is deeply held by policy makers, the general public, and scholars alike.

The most commonly assumed mechanism linking media use to obesity is reflected in the “couch-potato hypothesis,” whereby media use is related to obesity because it encourages inactivity, increases food consumption, or both. Despite widespread belief in the veracity of these mechanisms, few scholars have undertaken direct empirical assessment of such mediators, a weakness that has been noted by others [17,18]. Moreover, scholars have struggled to find strong and consistent links between television viewing and either physical activity or caloric intake [9,19-25]

## Other factors related to obesity and physical activity: the importance of friends

Within the voluminous literature aimed at identifying influences on youth obesity, friends have emerged as an important factor related to both obesity and physical activity [26]. There is a strong evidence that the social worlds of overweight and obese youth tend to be hostile, rejecting and negative [27-30]. Compared to normal-weight youth, children perceive overweight youth more negatively and are less likely to seek their company [31-33]. At the individual level, there is consistent evidence that overweight youth have fewer friends, are less likely to have mutual or reciprocated friendships, and are alone more frequently than normal-weight youth [30,34-36].

The relatively friendless state of overweight youth is unfortunate, because research also consistently show that time spent with friends is positively related to physical activity. A number of studies have found that children and adolescents are more physically active in the presence of friends than when alone, and that youth who report a greater presence of friends also report engaging in more physical activity [37,38]. Children who report being friendless and rejected by peers also report the least amount of physical activity [39,40]. Moreover, there is some evidence that overweight and non-overweight children may be equally physically active when in the presence of friends [36].

## Connecting youth obesity, television viewing, friends, and physical activity

To summarize, associations between television viewing and obesity among youth have proven to be unreliable, yet continue to appear sporadically in empirical studies. Overweight and obese children have fewer friends and spend less time with friends, and time spent with friends is positively related to physical activity. Though there is little evidence that television viewing decreases physical activity, there is evidence that time spent watching TV with friends is positively related to engaging in other activities with friends (i.e., youth with friends spend time with them, watching TV or otherwise [41].

Thus, despite the widespread belief that television viewing increases weight status, an equally plausible hypothesis is that overweight or obese youth spend less time with friends, which in turn is related to less time spent in physical activities, which they then fill with television viewing. Evidence indicates that a large proportion of television viewing occurs as a default when other options are not available [22,42]. This hypothesis suggests that television viewing may simply be a marker of the relatively meager social lives of overweight and obese youth.

The purpose of this paper is to examine the possibility that time with friends and physical activity are important mediators of the relationship between obesity and television viewing among older children and adolescents. Specifically, we posit that overweight/obese weight status will be negatively related to both time spent with friends and time spent in moderate-to-vigorous physical activity (MVPA). In turn, we expect time with friends to be positively related to time spent in MVPA, and that both time with friends and MVPA will be negatively related to time spent viewing television without friends. We hypothesize that overweight/obesity will be related to time spent viewing television without friends only indirectly via time spent with friends and time spent in MVPA.

## Methods

## Sample and procedures

We utilized data collected as part of the 2002 wave of the Child Development Supplement (CDS) to the Panel Study of Income Dynamics (PSID). The PSID has been an ongoing panel study since 1968, focusing primarily on the transfer of social and economic capital within families. In 2002, the CDS supplemented the core PSID with data focusing on family and child development collected via interview, parent-report, and child-report drawn from families who remained active in the PSID as of 2001. The CDS successfully reinterviewed 2017 families (91%) who provided data on health, cognitive, academic, and behavioral development, as well as time use from 2908 children and adolescents ages 5 to 18 [43].

Because the major variables of interest (overweight/obesity, physical activity, friendships and television viewing) and the relationships among them are known to vary between very young children and older children, we limited our sample to children and adolescents ages 10 to 18 years (n= 1,545, M Age = 13.81, SD = 2.55); 49% were boys; 45.8% were White, 42.8% were Black, 6.9% were Hispanic or Latino, and the remainder were Asian, Pacific Islander, Native American or Other race. Family median income was $48,800, with 15.1% of the families falling below poverty level. Parents had 12.90 (SD=2.62) years of education on average.

## Measures

### Child weight status

Weight and height information were collected via strain gauge lithium bath scales (Measurement Specialties, Inc. “Thinner MS-7” model; http://www.msiusa.com/default/index.asp) and measurement tape, respectively, during the home interview. Children were measured in stocking feet, in light clothing, with pockets emptied. BMI was calculated using the formula from the National Center for Chronic Disease Prevention and Health Promotion (weight [lbs] ÷ stature [in] ÷ stature [in] x 703), and converted to a BMI z-score using the Centers for Disease Control’s BMI growth reference to determine an age- and sex-specific BMI z-score for ages 2 to 20 years [44].

Child weight status is defined based on the 2000 Centers for Disease Control and Prevention growth reference for the United States [45]. Children are categorized as obese (BMI for age at or above the 95^th^ percentile), overweight (BMI for age at or above the 85^th^ percentile but less than the 95^th^ percentile of BMI for age), or normal weight (BMI for age is less than the 85^th^ percentile). In order to assess the association between overweight/obese status and other variables in the model, children with overweight or obese status were combined (coded 1) and compared to children of normal weight (coded 0).

### Time spent in activities: television viewing, time with friends & physical activity

Unique to the CDS are 24h time diaries logging the flow of children’s activities for 24 hours on one randomly chosen weekday and weekend day beginning at midnight. The diaries record the time every activity began and ended (duration of primary activities); where the child was; who was participating with the child; who was there but not participating; and what else the child was doing (secondary activities). Though always interviewer administered, diary activity reporters necessarily differ by child age. While younger children are incapable of self-report, not only are older children increasingly able to self-report, but they also need privacy to accurately do so. In 2002, The 36% of children who reported their activities without help from a primary caregiver averaged 14.48 years old; the 20% of children who reported their activities with caregiver help averaged 9.82 years old, and the 43% of children whose primary caregivers reported their activities averaged 7.68 years old. No diaries were administered solely to children under the age of 9. During consent/assent, all participants are informed that their answers are kept completely confidential and cannot be shared with any other participant. If completed, all diaries include a full accounting of activities over the 24 hour period. Participants in this study completed at least one time diary (98.3% completed both diaries, 0.5% completed a single weekend diary, and 1.3% completed a single weekday diary) [43]. There were no significant differences between participants who completed both time diaries versus those who completed either single day diaries (weekday or weekend).

#### Validity of time diary methods

An extensive body of research has demonstrated the validity and reliability of such diaries as representations of the way children and adults spend their time [46-48]. A large body of evidence demonstrates the validity and reliability of 24h time diaries as representations of the way children and adults spend their time [46,47,49,50]. Diary activities are generally highly correlated (on the order of .70 to .84) with direct observations [50] and substantially more accurate than self-report estimates of average daily or weekly time spent in activities [47,50,51]. In addition, there is evidence that parental report of their children’s TV viewing is more accurate in diaries than via parent or child self-report of average weekly estimates, and highly correlated with direct observational measures [52,53].

#### Representativeness of activities

Because 24h diaries are typically collected for 2 days, they are quite good at capturing daily activities, but less so for infrequent activities (e.g., volunteering). Unfortunately, asking respondents to keep 24h diaries for longer than 2 days results in precipitous drops in response rates (from around 80% to 40% or less for 3 or more days [48]), while adding considerable cost and respondent burden [54]. This may be less of a concern for viewing as most youth watch TV daily (even with the availability of newer media) [55,56]. CDS diary estimates of TV viewing are consistent with studies using weekly media diaries [57-59] and those based on representative samples in similar age ranges [60], fostering confidence in the accuracy of the diary estimates of TV viewing.

#### Time spent viewing television

Television viewing is represented as the number of hours per day children view television. In order to represent viewing in this way, the number of hours of viewing reported in the weekday diaries were multiplied by five, and the number of hours of viewing reported in the weekend diaries were multiplied by two. These products were then summed and divided by seven to arrive at a daily estimate of viewing per week using the following formula: [(sum of weekday hours x 5) + (sum of weekend hours x 2)]/7.

#### Time spent with friends

Time spent with friends is represented as the number of hours per day children spend in all activities with their friends. Because the CDS time diaries capture the social context of all activities (e.g., who, if anyone, was participating in activities with the child), we are able to capture the total amount of time children spend in all activities with their friends. Daily hours spent in activities where friends were present was calculated using the same formula presented for daily television viewing, above.

#### Moderate-to-vigorous physical activity (MVPA)

All activities in the time diaries were assigned metabolic equivalent (MET) scores utilizing the procedure advocated by Ainsworth et al. [61] to create measures of physical activity (PA). MET values are used to classify the relative energy cost of physical activities and describe activity patterns with one MET defined as the energy expended while sleeping [62,63]. MET values for activities published in the Compendium of Energy Expenditures for Youth [64] were assigned to activities by trained coders. Consensus was reached on all METs values before assignment. This procedure has been used to assess physical activity level (PAL) in the time diaries provided by the American Time Use Survey (ATUS) [65] and has been shown to provide a valid method for estimating energy expenditure in METS values, and time spent in adolescent MVPA [66-68]. Time spent in sedentary, light, moderate and vigorous activities were calculated based on duration and MET values for each activity. As with time spent viewing television and time spent with their friends, youth moderate-to-vigorous physical activity (MVPA) is represented as the numbers of hours per day spent in MVPA.

### Covariates

Model covariates included factors known to influence obesity, television viewing, physical activity, and/or time with friends. These included: (a) child age, (b) child gender, (c) child race/ethnicity, (d) family income-to-needs ratio (computed by dividing family income by the 2002 poverty threshold provided by the U.S. Census Bureau appropriate for family size), and (e) child maturational status

Because maturation-related misclassification may result in overestimations of overweight prevalence rates among early maturing adolescents and underestimations among later maturing adolescents [69,70], we utilized the Khamis-Roche (KR) method for predicting percent of adult stature [71]. Percent of adult stature has been shown to be significantly correlated with maturational status (range *r* = 0.50 to 0.70) and thus is a good proxy for maturational status when other measures (such as observational report of stages of pubertal development) are not available [72-74]. It is calculated from current stature (in), current weight (lb), and mid-parent stature (average height of both parents) (in). The regression equation for predicting adult stature takes the form: predicted adult stature = *β*_0_ + *β*_1_ stature + *β*_2_ weight + *β*_3_ mid-parent stature, where *β*_1_, *β*_2_, and *β*_3_ are the coefficients by which stature, weight, and mid-parent stature, respectively, are multiplied. Predicted adult stature / current stature then provides a measure of percent of adult stature at a given age.

## Statistical analysis

As the variables examined are observed in nature, we utilized path analysis to assess the fit of the model to the data. Path analysis has two advantages over linear regression for our purposes. First, because our proposed model is not fully-saturated, path analysis allows assessment of model-fit (in contrast to regression models which are fully-saturated by default). Second, path coefficients are estimated via simultaneous equation estimation, thus adjusting standard errors for the number of equations computed [75]. We utilized full-information-maximum-likelihood estimation with robust standard errors, a procedure that provides robust estimates utilizing all available data and accounts for non-independence within families [76]. Model fit was assessed using the combination of fit indices recommended by Bollen (1989), including (a) the Chi-square statistic, (b) the Comparative Fit Index (CFI), (c) the Tucker-Lewis index (TLI), and (d), the Root Mean Square Error of Approximation (RMSEA). Because Chi-square is highly sensitive in large samples, minimally acceptable fit includes the combination of CFI and TLI estimates greater than .95 and RMSEA estimates of .05 or below. Very good fit is indicated by the combination of a non-significant Chi-square test (p<.05), CFI and TLI estimates of .98 or above, and RMSEA estimates of .03 or below.

Mediation was assessed by computing estimates of direct, indirect, and total effects of the associations specified in the model using full information maximum likelihood with robust standard errors. Direct effects represent associations between variables unmediated by any other variable in the model. Indirect effects represent mediated effects (or combined mediated effects for paths through multiple mediators). Total effects are the sum of the direct and indirect effects. In order to examine specific mediated effects when indirect effects represent combined mediated effects (for example the mediated effect of overweight/obese on time spent television via time spent with friends only), we conducted product-of-coefficient tests [77]. This consists of (1) estimating the effect of overweight/obesity on potential mediators (α coefficient), (2) estimating the independent effect of the potential mediators on television viewing (β coefficient), and (3) computing the mediated effect (the product of the αβ coefficients). This mediated effect represents the effect of obesity on television viewing via the proposed mediators in units of television viewing (the outcome). Because significant mediation can exist even in the presence of non-significant direct effects, both single and multiple mediator models were assessed [78]. Bootstrapping resampling techniques were used to estimate standard errors and confidence intervals of mediated effects [77,79].

Analyses were conducted in Stata 13.1. Covariates (Child Age, Child Gender, Child Race, Child Maturational Status, and Family Income-to-Needs Ratio), were included in path models as correlated exogenous variables predicting all other variables in the model as suggested by Bollen [75], and in mediator analyses [77,78].

## Results

### Preliminary results

Descriptive statistics are presented in Table 1. Twenty-one percent of youth were classified as obese, 16% as overweight, and 62% as normal weight, a finding generally in-line with current estimates [1,80]. Overall, youth watched an average of 2.62 hours of television per week. This estimate is consistent with existing studies using weekly media diaries [57-59]. It is interesting to note that much more of this viewing occurred without friends (M=2.32) than with friends (M=.28, or about 17 minutes per week). It also worth noting that youth spent almost 2 hours per week with their friends, but only 45 minutes per week, on average, in moderate-to-vigorous physical activity. Zero-order correlations among all variables utilized in the path analyses are presented in Table 2.

**Table 1:** Sample Characteristics (N=1,545)

Variables	Mean (*SD*) or %
Child Age, years	13.81 (2.55)
Boys	49 %
Child Race	
Non-Hispanic White	46 %
Non-Hispanic Black	43 %
Hispanic or Latino	7 %
Other (Asian, Pacific islander, Native American, Other Race)	4 %
Family Income	$ 68,149 (93,867)
Income-to-Needs Ratio	3.80 (5.34)
Parental Education, years	12.90 (2.62)
Child Weight Status	
Child Normal Weight	62 %
Child Overweight	16.1 %
Child Obese	21.9 %
Time Spent with Friends, hours per week	1.88 (2.19)
Moderate-to-Vigorous Activity, hours per week	.75 (1.05)
Total TV Viewing, hours per week	2.62 (1.83)
Television Viewing with Friends, hours per week	.28 (.69)
Television Viewing without Friends, hours per week	2.32 (1.77)

**Table 2:** Correlation matrix for all variables in the model

	1	2	3	4	5	6	7	8
1. Child Age								
2. Child Gender^a^	-0.06							
3. Child Ethnicity^b^	-0.06	-0.03						
4. Income-to-Needs Ratio	0.03	-0.04	0.22					
5. Child Maturational Status	0.80	-0.18	-0.09	0.02				
6. Overweight & Obese^c^	-0.04	0.02	-0.16	-0.05	-0.02			
7. Time Spent with Friends^d^	0.26	-0.06	0.06	0.04	0.23	-0.11		
8. Moderate-to-vigorous Activity^d^	-0.04	0.21	0.07	-0.01	-0.05	-0.05	0.13	
9. Television Viewing without Friends^d^	-0.05	0.05	-0.11	-0.08	-0.04	0.12	-0.29	-0.09

### Path analyses results

**Figure 1:**
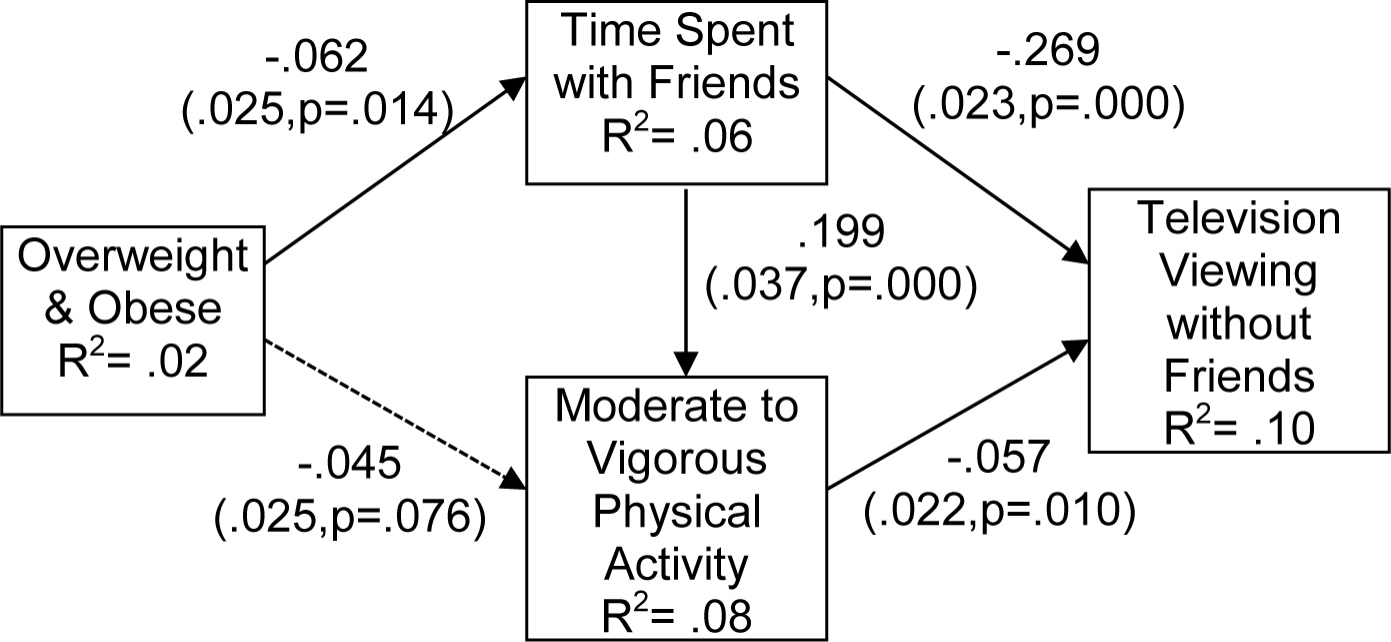
Model 1: Proposed Mediation Model Linking Obesity to Television Viewing via Time Spent with Friends *Note*. Path coefficients presented in standardized form; Robust standard errors and exact p values for each coefficient are presented in parentheses. Dotted lines represent non-significant paths; Estimates of Model Fit: χ^2^= 13.45, df = 6, p=.036, CFI = .98, TFI = .92, RMSEA = .05.

**Figure 2:**
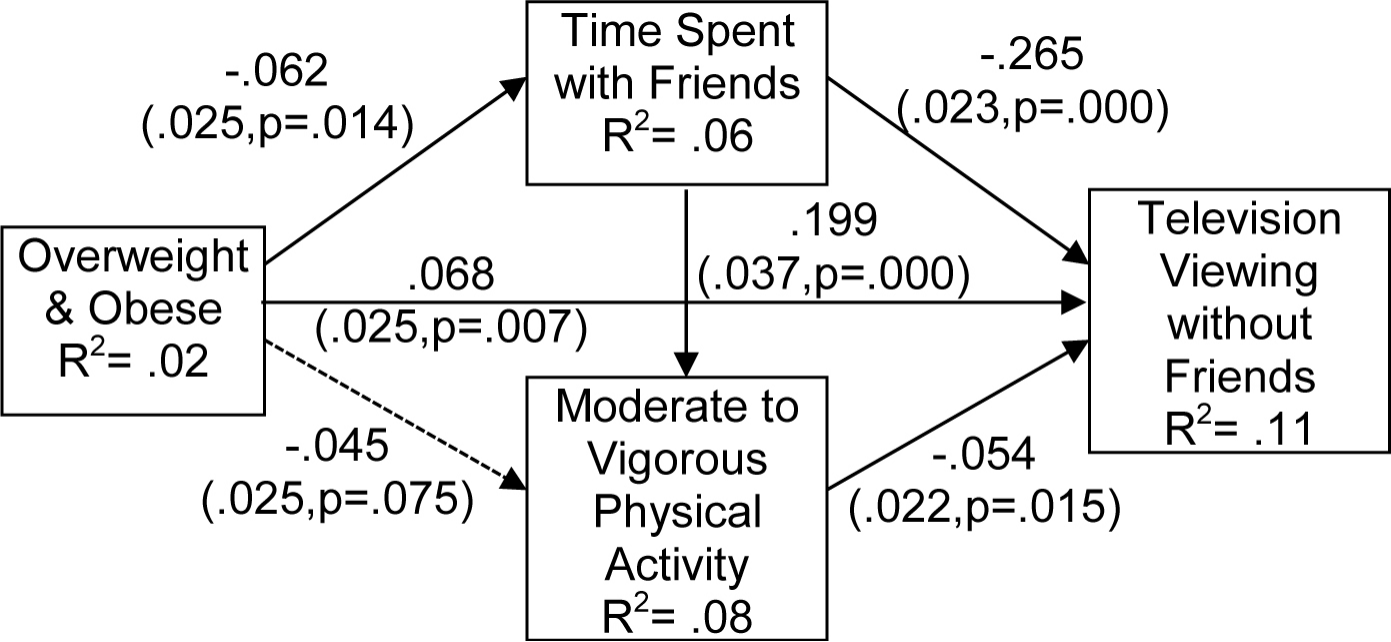
Model 2: Final Mediation Model Linking Obesity to Television Viewing via Time Spent with Friends *Note*. Path coefficients presented in standardized form; Robust standard errors and exact p values for each coefficient are presented in parentheses. Dotted lines represent non-significant paths; Estimates of Model Fit: χ^2^= 5.77, df = 5, p=.33, CFI = .99, TFI = .99, RMSEA = .01.

Standardized path coefficient estimates for our hypothesized model whereby overweight/obesity is only indirectly related to time spent television viewing via time spent with friends and time spent in MVPA are presented in figure 1. Our hypothesized model (Model 1) had only minimally acceptable fit with the data (χ^2^= 13.45, df = 6, p=.036, CFI = .98, TFI = .92, RMSEA = .05), indicating that important relationships were omitted. Therefore, we estimated a second model (Model 2) which included a direct relationship between obesity and television viewing, while identical to Model 1 in all other aspects. The standardized path estimates for Model 2 are presented in Figure 2. Model 2 fit the data extremely well (χ^2^= 5.77, df = 5, p=.33, CFI = .99, TFI = .99, RMSEA = .01), and significantly better than Model 1, our proposed model (Δχ^2^= 7.68, df = 1, p=.01) [75].

All of the path coefficients were in the expected direction in both models. However, regardless of significance, the path coefficients tended to be somewhat small. It should also be noted that despite its excellent fit statistics, Model 2 explained 10% of the variance in time spent television viewing (R^2^=.102). When the variance explained by covariates is removed, Model 2 explained 8% of the variance in television viewing (R^2^=.079). The magnitude of the effects found in this study echo those of existing studies examining obesity and television viewing in youth, in which small to extremely small effect sizes are the norm rather than the exception [6-9,16].

However, there are two notable exceptions in Model 2 (Figure 2). Time spent with friends was negatively related to time spent viewing television without friends (β=-.26), and positively related to time spent in MVPA (β =.20). The relationship between time spent with friends and physical activity is well-established [37-40], and thus unsurprising. However, friendship measures of any kind are scarce to non-existent in the voluminous literature examining associations between obesity and screen media among youth [26]. Our findings suggest that the omission of friendship indicators may be hindering more nuanced understandings of the relationship between television viewing and youth obesity.

### Assessment of mediation

Estimates of the direct, indirect and total effects from model 2 are presented in Table 3. Blank cells in Table 3 represent associations which were not specified in model 2. As noted, direct effects represent unmediated associations between variables, while indirect effects represent combined mediated effects (e.g., the indirect relationship between variables via all specified mediators combined). All direct associations examined in the model were significant except one – overweight/obese status was not directly related to time spent in MVPA, a finding already reflected in Figure 2. The indirect relationship between overweight/obesity and time spent in MVPA via time spent with friends was significant, as was the indirect relationship between overweight/obesity and time spent watching television. The proportion of the relationship between overweight/obesity and MVPA mediated by time spent with friends was .21, while the proportion of the relationship between overweight/obesity and television viewing mediated by time spent with friends and MVPA combined was .22. This suggests that mediated associations between overweight/obesity and television viewing were driven almost entirely by time spent with friends. This conclusion is further supported by product-of-coefficient tests estimating mediation between overweight/obesity and television viewing via time spent with friends and time spent in MVPA separately. These analyses indicated that the mediated relationship of overweight/obesity and television via time with friends was .057 (SE = .011, p=.014, 95% CI .03-.08) while the mediated relationship of overweight/obesity and television via time spent in MVPA was .013 (SE = .014, p=.231, 95% CI -.13-.04).

**Table 3:** Direct, indirect, and total effect estimates of overweight/obese status, time spent with friends, and time spent in MVPA on time spent television viewing.

	**Model Outcomes**								
	Time with Friends			Time in MVPA			Time Viewing TV without Friends		
	Coefficient (SE)	p value	95% CI	Coefficient (SE)	p value	95% CI	Coefficient (SE)	p value	95% CI
**Direct Effects:**									
Overweight/Obese	-0.279 (0.114)	0.015	(-0.50, -0.06)	-0.096 (0.054)	0.074	(-0.20, 0.01)	0.246 (0.093)	0.008	(0.06, 0.42)
Time in MVPA	--	--	--	--	--	--	-0.091 (0.038)	0.017	(-0.17, -0.02)
Time with Friends	--	--	--	0.095 (0.019)	0.000	(0.05, 0.13)	-0.214 (0.020)	0.000	(-0.25, -0.18)
**Indirect Effects:**									
Overweight/Obese	0	0	0	-0.027 (0.011)	0.015	(-0.05, -0.01)	0.071 (0.026)	0.006	(0.02, 0.12)
Time in MVPA	--	--	--	--	--	--	0	0	0
Time with Friends	--	--	--	0	0	0	-0.009 (0.002)	0.000	(-0.012, -0.005)
**Total Effects:**									
Overweight/Obese	-0.279 (0.114)	0.015	(-0.50, -0.06)	-0.123 (0.055)	0.026	(-0.23, -0.01)	0.317 (0.097)	0.001	(0.13, 0.51)
Time in MVPA	--	--	--	--	--	--	-0.091 (0.038)	0.017	(-0.17, -0.02)
Time with Friends	--	--	--	0.095 (0.019)	0.000	(0.06, 0.13)	-0.222 (0.019)	0.000	(-0.26, -0.18)

*Note*. Effect coefficients are presented in unstandardized form.

## Discussion

In this study we examined an alternative to the often proposed notion that television viewing increases childhood obesity via increased caloric intake, decreased physical activity, or both. Existing evidence of associations among friendship, physical activity and youth obesity [9,19-25], as well as evidence linking these factors to television viewing served as the bases for our proposal that obese youth, who have fewer friends and are thus less active, tend to watch more television as a default activity (Model 1).

Overall, we found reasonable evidence to suggest that elevated weight status may well be related to television viewing through social factors such as friendships. Overweight/obese status was negatively related to time spent with friends, which in turn was positively related to time spent in MVPA and negatively related to television viewing without friends. Time spent in MVPA was negatively related to television viewing without friends. Time spent with friends significantly mediated the relationship between overweight/obesity and television viewing. On the other hand, Overweight/obesity was not related to time spent in MVPA, nor was there a mediated effect of MVPA on the relationship between overweight/obesity and time spent viewing television.

However, we also found that overweight/obesity remained directly related to television viewing without friends, over and above any indirect effects via either time with friends or time spent in MVPA. This suggests that some important but as yet unidentified mechanism linking obesity to television viewing may be operating. Given that our measure of television viewing reflected viewing without friends present, it seems likely that the direct relationship between obesity and viewing without friends may reflect an aspect of friendship not captured in these data.

## Limitations

There are several limitations worth noting. First, as the data were all drawn from the same time period, temporal or causal associations cannot be assessed. We view these findings as a first step to assessing more fully how the associations among obesity, friendships, physical activity and television viewing unfold overtime. Second, in this paper we focus specifically on time spent television viewing for two reasons, (1) the vast majority of existing research in this area focuses specifically on associations between youth obesity and television viewing [81,82], and (2) despite media reports and popular opinion, television viewing still occupies the largest amount of time youth spend with screen media [83,84]. However, it is clear that youth are also engaged in other forms of screen media, including those on mobile platforms [83]. Preliminary analyses examining the proposed model with either time spent playing videos games or computer use indicated large variation between models including each type of screen media, differences found by others as well [85-87]. Thus, while we believe that examining models including various types of media use is important and needed, these multiple examinations are beyond the scope of this paper, and will be reserved for future work. Moreover, the year these data were collected (2002) does not allow us to examine newer forms of media popular with youth, such as mobile devices and social media. This will be an important area for future inquiry. In addition, we examined only one aspect of friendships-hours per week that youth spend time with friends. There is evidence to suggest that other aspects of friendships, such as the quality of youth friendships and perceptions of social support by friends, are related to both childhood overweight and obesity [26,35,88,89]. This is also true of peer group factors, such as experiences in school [90-92]. Thus, it will be important to assess exactly how these different aspects of social relationships may influence associations between obesity and media use, and the ways in which these influences unfold over time as children develop. Finally, because gender, age, ethnicity and SES were treated as covariates in these analyses, it will be important to assess the extent to which associations might vary by these factors (by gender or developmental stage for example) in future research.

## Conclusions

Despite a great deal of empirical work, evidence for the hypothesis that television must cause elevated weight status via physical activity or caloric intake has proven remarkably elusive [9,19-25]. For example, there is little evidence supporting the notion that physical activity is importantly related to television viewing [23], a finding replicated in this study. Additionally, empirical assessment of the mediating effects of physical activity or caloric intake on associations between media use and youth obesity are surprisingly scarce [93]. We would like to call attention again to the fact that the magnitude of associations between overweight/obesity and television viewing, as well as the proportion of variance explained in this study were fairly small. Though rarely discussed, small (though significant) associations between weight status and television viewing in youth is quite common in this literature, as evidenced by several meta-analyses [81,82].

Taken together, our results indicate that including friendship measures is important for future attempts to “unpack” relationships between obesity and television viewing. Our findings do not negate the possibility that television viewing is linked to obesity via other mechanisms such as increased caloric intake. Rather, they underscore the importance of moving from examinations of simple or bivariate relationships between television viewing and weight status, to models aimed at identifying key mechanisms mediating their relationship. They also underscore the importance of considering other pathways, such as friendships, through which screen media might be related to obesity. Finally, our results suggest the possibility that interventions focused on youth obesity might benefit from re-thinking the pervasive commitment to decreasing screen-time via careful attention to peer and friendship dynamics.

## List of abbreviations used

CDS: Child Development Supplement; PSID: Panel Study of Income Dynamics; BMI: body mass index; NHANES: National Health and Nutrition Examination Surveys; MET: metabolic equivalent of task; PA: physical activity; PAL: physical activity level; MVPA: moderate-to-vigorous physical activity; ATUS: American Time Use Survey; CRPBI: Child Report of Parent Behavior Inventory

## Competing interests

The authors declare that they do not have competing interests.

## Authors’ contributions

EV and SEP conducted the statistical analysis and interpretation of results, and contributed to the writing and editing of the manuscript. EH and HC contributed to the writing and editing of the manuscript. All authors have read and approved the final manuscript.

## Declarations

This article has been published as part of *International Journal of Behavioral Nutrition and Physical Activity* Volume 12 Supplement 1, 2015: The science of childhood obesity: an individual to societal framework. The full contents of the supplement are available online at http://www.ijbnpa.org/supplements/12/S1. Publication costs for this supplement were funded by the Michael & Susan Dell Foundation.

## References

[B1] Ogden CL Carroll MD Kit BK Flegal KM Prevalence of childhood and adult obesity in the United States, 2011-2012 JAMA 2014 311 8 806 814 10.1001/jama.2014.732 24570244 PMC4770258

[B2] Daniels SR Arnett DK Eckel RH Gidding SS Hayman LL Kumanyika S Overweight in children and adolescents pathophysiology, consequences, prevention, and treatment Circulation 2005 111 15 1999 2012 10.1161/01.CIR.0000161369.71722.10 15837955

[B3] Must A Spadano J Coakley EH Field AE Colditz G Dietz WH The disease burden associated with overweight and obesity JAMA 1999 282 16 1523 1529 10.1001/jama.282.16.1523 10546691

[B4] Dietz WH Gortmaker SL Do we fatten our children at the television set? Obesity and television viewing in children and adolescents Pediatrics 1985 75 5 807 12 3873060

[B5] Davison KK Marshall SJ Birch LL Cross-sectional and longitudinal associations between TV viewing and girls’ body mass index, overweight status, and percentage of body fat J Pediatr 2006 149 1 32 37 10.1016/j.jpeds.2006.02.003 16860123 PMC2562303

[B6] Mitchell JA Pate RR Liese AD Changes in cardiovascular disease risk factors from age 9 to 19 and the influence of television viewing Obesity 2013 21 2 386 393 10.1002/oby.20014 23404755

[B7] Pérez A Hoelscher DM Springer AE Brown HS Kelder SH Barroso CS Peer Reviewed: Physical Activity, Watching Television, and the Risk of Obesity in Students, Texas, 2004-2005 Preventing Chronic Disease 2011 8 3 A61 21477501 PMC3103566

[B8] Robinson TN Chen HN Killen JD Television and music video exposure and risk of adolescent alcohol use Pediatrics 1998 102 5 1 6 9794984 10.1542/peds.102.5.e54

[B9] Robinson TN Killen JD Ethnic and gender differences in the relationships between television viewing and obesity, physical activity, and dietary fat intake Journal of Health Education 1995 26 Suppl 2 S91 S98

[B10] Hendrix KS Carroll AE Downs SM Screen Exposure and Body Mass Index Status in 2- to 11-Year-Old Children Clinical Pediatrics 2014 53 6 593 600 10.1177/0009922814526973 24634433 PMC8905453

[B11] Robinson TN Hammer LD Wilson DM Killen JD Kraemer HC Hayward C Does television viewing increase obesity and reduce physical activity? Cross-sectional and longitudinal analyses among adolescent girls Pediatrics 1993 91 2 273 80 8424000

[B12] DuRant RH Baranowski T Johnson M Thompson WO The relationship among television watching, physical activity, and body composition of young children Pediatrics 1994 94 4 449 455 7936851

[B13] Steffen LM Dai S Fulton JE Labarthe DR Overweight in children and adolescents associated with TV viewing and parental weight: Project HeartBeat! Am J Prev Med 2009 37 1 S50 S55 10.1016/j.amepre.2009.04.017 19524156 PMC2746249

[B14] McMurray RG Harrell JS Deng S Bradley CB Cox LM Bangdiwala SI The influence of physical activity, socioeconomic status, and ethnicity on the weight status of adolescents Obesity research 2000 8 2 130 139 10.1038/oby.2000.14 10757199

[B15] Wake M Hesketh K Waters E Television, computer use and body mass index in Australian primary school children Journal of Paediatrics and Child Health 2003 39 2 130 134 10.1046/j.1440-1754.2003.00104.x 12603802

[B16] Marshall SJ Biddle SJH Gorely T Cameron N Murdey I Relationships between media use, body fatness and physical activity in children and youth: a meta-analysis Int J Obes Relat Metab Disord 2004 28 10 1238 1246 10.1038/sj.ijo.0802706 15314635

[B17] Brown H Hume C Pearson N Salmon J A systematic review of intervention effects on potential mediators of children’s physical activity BMC Public Health 2013 13 1 165 10.1186/1471-2458-13-165 23433143 PMC3585884

[B18] Lubans DR Foster C Biddle SJ A review of mediators of behavior in interventions to promote physical activity among children and adolescents Preventive Medicine 2008 47 5 463 470 10.1016/j.ypmed.2008.07.011 18708086

[B19] Anderson PM Butcher KF Childhood obesity: trends and potential causes The Future of Children 2006 16 1 19 45 10.1353/foc.2006.0001 16532657

[B20] Sallis JF Prochaska JJ Taylor WC A review of correlates of physical activity of children and adolescents Medicine and Science in Sports and Exercise 2000 32 5 963 975 10795788 10.1097/00005768-200005000-00014

[B21] Waller CE Du S Popkin BM Patterns of overweight, inactivity, and snacking in Chinese children Obesity Research 2003 11 8 957 961 10.1038/oby.2003.132 12917500

[B22] Vandewater EA Bickham DS Lee JH Time well spent? Relating media use to children’s free-time activities Pediatrics 2006 117 2 e181 e185 10.1542/peds.2005-0812 16452327 PMC2862999

[B23] Hager RL Television Viewing and Physical Activity in Children Journal of Adolescent Health 2006 39 5 656 661 10.1016/j.jadohealth.2006.04.020 17046501

[B24] Pearson N Braithwaite R Biddle S Sluijs E Atkin A Associations between sedentary behaviour and physical activity in children and adolescents: a meta‐analysis Obesity Reviews 2014 15 8 666 675 10.1111/obr.12188 24844784 PMC4282352

[B25] Must A Bandini LG Tybor DJ Phillips SM Naumova EN Dietz WH Activity, Inactivity, and Screen Time in Relation to Weight and Fatness Over Adolescence in Girls Obesity (Silver Spring) 2007 15 7 1774 1781 10.1038/oby.2007.211 17636096

[B26] Salvy S Bowker J Peers and Obesity during Childhood and Adolescence: A Review of the Empirical Research on Peers, Eating, and Physical Activity J Obes Weight Loss Ther 2014 4 207 2 10.4172/2165-7904.1000207 PMC5228616 28090396

[B27] Hayden‐Wade HA Stein RI Ghaderi A Saelens BE Zabinski MF Wilfley DE Prevalence, Characteristics, and Correlates of Teasing Experiences among Overweight Children vs Obesity Research 2005 13 8 1381 1392 10.1038/oby.2005.167 16129720

[B28] Adams RE Bukowski WM Peer victimization as a predictor of depression and body mass index in obese and non‐obese adolescents Journal of Child Psychology and Psychiatry 2008 49 8 858 866 10.1111/j.1469-7610.2008.01886.x 18355219

[B29] Storch EA Milsom VA DeBraganza N Lewin AB Geffken GR Silverstein JH Peer victimization, psychosocial adjustment, and physical activity in overweight and at-risk-for-overweight youth Journal of Pediatric Psychology 2007 32 1 80 89 16601255 10.1093/jpepsy/jsj113

[B30] Strauss RS Pollack HA Social marginalization of overweight children Archives of Pediatrics & Adolescent Medicine 2003 157 8 746 752 10.1001/archpedi.157.8.746 12912779

[B31] Bell SK Morgan SB Children’s attitudes and behavioral intentions toward a peer presented as obese: Does a medical explanation for the obesity make a difference? Journal of Pediatric Psychology 2000 25 3 137 145 10.1093/jpepsy/25.3.137 10780140

[B32] Sigelman CK The effect of causal information on peer perceptions of children with physical problems Journal of Applied Developmental Psychology 1991 12 2 237 253 10.1016/0193-3973(91)90014-U

[B33] Zeller MH Reiter‐Purtill J Ramey C Negative peer perceptions of obese children in the classroom environment Obesity 2008 16 4 755 762 10.1038/oby.2008.4 18379560 PMC2713023

[B34] Valente TW Fujimoto K Chou CP Spruijt-Metz D Adolescent Affiliations and Adiposity: A Social Network Analysis of Friendships and Obesity Journal of Adolescent Health 2009 45 2 202 204 10.1016/j.jadohealth.2009.01.007 19628148 PMC2747768

[B35] Pedersen S Vitaro F Barker ED Borge AI The timing of Middle‐Childhood peer rejection and friendship: Linking early behavior to Early‐Adolescent adjustment Child Development 2007 78 4 1037 1051 10.1111/j.1467-8624.2007.01051.x 17650124

[B36] Salvy S-J Coelho JS Kieffer E Epstein LH Effects of social contexts on overweight and normal-weight children’s food intake Physiol Behavior 2007 92 5 840 846 10.1016/j.physbeh.2007.06.014 PMC2725404 17628616

[B37] Beets MW Vogel R Forlaw L Pitetti KH Cardinal BJ Social support and youth physical activity: the role of provider and type American Journal of Health Behavior 2006 30 3 278 289 16712442 10.5555/ajhb.2006.30.3.278

[B38] Duncan SC The role of cognitive appraisal and friendship provisions in adolescents’ affect and motivation toward activity in physical education Research Quarterly for Exercise and Sport 1993 64 3 314 323 10.1080/02701367.1993.10608816 8235053

[B39] Keresztes N Piko BF Pluhar ZF Page RM Social influences in sports activity among adolescents J R Soc Promot Health 2008 128 1 21 25 10.1177/1466424007085228 18274326

[B40] Barkley JE Salvy SJ Roemmich JN The effect of simulated ostracism on physical activity behavior in children Pediatrics 2012 129 3 e659 e666 10.1542/peds.2011-0496 22311997

[B41] Bickham DS Rich M Is television viewing associated with social isolation?: roles of exposure time, viewing context, and violent content Arch Pediatr Adolesc Med 2006 160 4 387 392 10.1001/archpedi.160.4.387 16585484

[B42] Huston AC Wright JC Sigel IE, Renninger KA Mass media and children’s development Handbook of Child Psychology 1997 John Wiley & Sons 999 1058

[B43] McGonagle KA Sastry N Cohort Profile: The Panel Study of Income Dynamics’ Child Development Supplement and Transition into Adulthood Study Int J Epidemiol 2014 10.1093/ije/dyu076 PMC4553706 24706732

[B44] Ogden CL Kuczmarski RJ Flegal KM Mei Z Guo S Wei R Centers for Disease Control and Prevention 2000 growth charts for the United States: improvements to the 1977 National Center for Health Statistics version Pediatrics 2002 109 1 45 60 10.1542/peds.109.1.45 11773541

[B45] Kuczmarski RJ Ogden CL Guo SS Grummer-Strawn LM Flegal KM Mei Z 2000 CDC Growth Charts for the United States: methods and development Vital Health Stat 11 2002 246 1 190 12043359

[B46] Juster FT Stafford FP The allocation of time: Empirical findings, behavioral models, and problems of measurement Journal of Economic Literature 1991 29 471 522

[B47] Juster FT Ono H Stafford FP An assessment of alternative approaches to the measures of time use Sociological Methodology 2003 33 19 54 10.1111/j.0081-1750.2003.t01-1-00126.x

[B48] Robinson JP Godbey G Time for Life: The Surprising Ways Americans Use Their Time 1997 University Park, Pennsylvania: The Pennsylvania State University Press

[B49] Juster FT Response errors in the measurement of time use Journal of the American Statistical Association 1986 81 390 402 10.1080/01621459.1986.10478283

[B50] Robinson JP Juster FT, Stafford FP The validity and reliability of diaries versus alternative time use measures Time, Goods, and Well-being 1985 Ann Arbor, MI: Institute for Social Research, University of Michigan 33 62

[B51] Hunt E McKay EA What Can Be Learned From Adolescent Time Diary Research Journal of Adolescent Health 2014 56 3 259 266 25592884 10.1016/j.jadohealth.2014.11.007

[B52] Anderson DR Field DE Collins PA Lorch EP Natham JG Estimates of young children’s time with television: A methodological comparison of parent reports with time-lapse video home observation Child Development 1985 56 5 1345 1357 10.2307/1130249 4053746

[B53] Robinson JL Winiewicz DD Fuerch JH Roemmich JN Epstein LH Relationship between parental estimate and an objective measure of child television watching The International Journal of Behavioral Nutrition and Physical Activity 2006 3 43 10.1186/1479-5868-3-43 17129381 PMC1687199

[B54] Bianchi S Robinson J What did you do today? Children’s use of time, family composition, and the acquisition of social capital Journal of Marriage and the Family 1997 59 2 332 44 10.2307/353474

[B55] Rideout VJ Foehr UG Roberts DF Generation M2: Media in the lives of 8-18 year-olds 2010 Menlo Park, CA: The Henry J. Kaiser Family Foundation

[B56] Vandewater EA Denis LM Media, social networking, and pediatric obesity Pediatric Clinics of North America 2011 58 6 1509 1519 10.1016/j.pcl.2011.09.012 22093866 PMC5737742

[B57] Roberts D Foehr U Rideout V Generation M: Media in the Lives of 8-18 Year-olds A Kaiser Family Foundation Study 2005 Menlo Park, CA: The Henry J. Kaiser Family Foundation March

[B58] Anderson DR Huston AC Schmitt KL Linebarger DL Wright JC Early childhood television viewing and adolescent behavior: The recontact study Monographs of the Society for Research in Child Development 2001 66 1 1 147 10.1111/1540-5834.00121 11326591

[B59] Huston AC Wright JC Marquis J Green SB How young children spend their time: Television and other activities Developmental Psychology 1999 35 4 912 925 10442861 10.1037//0012-1649.35.4.912

[B60] Roberts DF Foehr UG Kids & Media in America 2004 New York, NY: Cambridge University Press

[B61] Ainsworth BE Haskell WL Whitt MC Irwin ML Swartz AM Strath SJ Compendium of physical activities: An update of activity codes and MET intensities Medicine and Science in Sports and Exercise 2000 32 8 Suppl S498 S504 10993420 10.1097/00005768-200009001-00009

[B62] Spadano JL Must A Bandini LG Dallal GE Dietz WH Energy cost of physical activities in 12-y-old girls: MET values and the influence of body weight International Journal of Obesity 2003 27 12 1528 1533 10.1038/sj.ijo.0802440 14634685

[B63] Salmon J Okely AD Physical activity in young people--Assessment and methodological issues Journal of Science and Medicine in Sport 2009 12 5 513 514 10.1016/j.jsams.2009.04.001 19443268

[B64] Ridley K Ainsworth BE Olds TS Development of a compendium of energy expenditures for youth The International Journal of Behavioral Nutrition and Physical Activity 2008 5 45 10.1186/1479-5868-5-45 18782458 PMC2564974

[B65] Tudor-Locke C Washington TL Ainsworth BE Troiano RP Linking the American Time Use Survey (ATUS) and the Compendium of Physical Activities: Methods and rationale Journal Of Physical Activity & Health 2009 6 3 347 353 19564664 10.1123/jpah.6.3.347

[B66] Bratteby LE Sandhagen B Samuleson G Physical activity, energy expenditure and their correlates in two cohorts of Swedish subjects between adolescence and early adulthood European Journal of Clinical Nutrition 2005 59 11 1324 1334 10.1038/sj.ejcn.1602246 16091767

[B67] Ekelund U Assessment of physical activity and energy expenditure in adolescents 2002 Stockholm, Sweden: Karolinska University Press

[B68] Ekelund U Brage S Wareham NJ Physical activity in young children The Lancet 2004 363 9415 1163 10.1016/S0140-6736(04)15910-2 15064045

[B69] Himes JH Maturation‐related deviations and misclassification of stature and weight in adolescence American Journal of Human Biology 1999 11 4 499 504 10.1002/(SICI)1520-6300(1999)11:4<499::AID-AJHB9>3.0.CO;2-M 11533969

[B70] Wang Y Is obesity associated with early sexual maturation? A comparison of the association in American boys versus girls Pediatrics 2002 110 5 903 910 10.1542/peds.110.5.903 12415028

[B71] Khamis HJ Roche AF Predicting adult stature without using skeletal age: the Khamis-Roche method Pediatrics 1994 94 4 504 507 7936860

[B72] Beunen GP Malina RM Lefevre J Claessens AL Renson R Simons J Prediction of adult stature and noninvasive assessment of biological maturation Medicine and Science in Sports and Exercise 1997 29 2 225 230 10.1097/00005768-199702000-00010 9044227

[B73] Himes JH Obarzanek E Baranowski T Wilson DM Rochon J McClanahan BS Early Sexual Maturation, Body Composition, and Obesity in African‐American Girls Obesity Research 2004 12 Suppl 64S 72S 15489469 10.1038/oby.2004.270

[B74] Roche AF Tyleshevski F Rogers E Non-invasive measurements of physical maturity in children Research Quarterly for Exercise and Sport 1983 54 4 364 371 10.1080/02701367.1983.10605321

[B75] Bollen KA Structural Equations with Latent Variables Wiley-Interscience 1989

[B76] Schafer J Graham JW Missing data: Our view of the state of the art Psychological Methods 2002 7 2 147 177 12090408

[B77] Cerin E MacKinnon DP A commentary on current practice in mediating variable analyses in behavioural nutrition and physical activity Public Health Nutrition 2009 12 8 1182 1188 10.1017/S1368980008003649 18778534 PMC4207270

[B78] MacKinnon DP Fairchild AJ Fritz MS Mediation analysis Annual Review of Psychology 2007 58 593 614 10.1146/annurev.psych.58.110405.085542 16968208 PMC2819368

[B79] Pituch KA Lee Y-k The influence of system characteristics on e-learning use Computers & Education 2006 47 2 222 244 10.1016/j.compedu.2004.10.007

[B80] Xi B Mi J Zhao M Zhang T Jia C Li J Trends in Abdominal Obesity Among US Children and Adolescents Pediatrics 2014 134 2 e334 e339 10.1542/peds.2014-0970 25049347

[B81] Dennison BA Edmunds LS The role of television in childhood obesity Progress in Pediatric Cardiology 2008 25 2 191 197 10.1016/j.ppedcard.2008.05.010

[B82] Jordan AB Heavy television viewing and childhood obesity Journal of Children and Media 2007 1 1 45 54 10.1080/17482790601005124

[B83] Rideout VJ Foehr UG Roberts DF Generation M [superscript 2]: Media in the Lives of 8-to 18-Year-Olds Henry J Kaiser Family Foundation 2010

[B84] Roberts DF Foehr UG Rideout VJ Generation M: Media in the lives of 8-18 year-olds Henry J. Kaiser Family Foundation 2005

[B85] Rey-Lopez JP Vicente-Rodreguez G Biosca M Moreno LA Sedentary behaviour and obesity development in children and adolescents Nutrition, Metabolism and Cardiovascular Diseases 2008 18 3 242 251 10.1016/j.numecd.2007.07.008 18083016

[B86] De Jong E Visscher T Hirasing R Heymans M Seidell J Renders C Association between TV viewing, computer use and overweight, determinants and competing activities of screen time in 4-to 13-year-old children International Journal of Obesity 2011 37 1 47 53 22158265 10.1038/ijo.2011.244

[B87] Vandewater EA Shim M-S Caplovitz AG Linking obesity and activity level with children’s television and video game use Journal of Adolescence 2004 27 1 71 85 10.1016/j.adolescence.2003.10.003 15013261

[B88] Lawman HG Wilson DK Associations of social and environmental supports with sedentary behavior, light and moderate-to-vigorous physical activity in obese underserved adolescents The International Journal of Behavioral Nutrition and Physical Activity 2014 11 1 92 10.1186/s12966-014-0092-1 25163029 PMC4145237

[B89] Schaefer DR Simpkins SD Using Social Network Analysis to Clarify the Role of Obesity in Selection of Adolescent Friends American Journal of Public Health 2014 104 7 1223 9 10.2105/AJPH.2013.301768 24832139 PMC4056220

[B90] Zeller MH Boles RE Reiter-Purtill J The additive and interactive effects of parenting style and temperament in obese youth seeking treatment International Journal of Obesity 2008 32 10 1474 1480 10.1038/ijo.2008.125 18698318 PMC3541051

[B91] van Geel M Vedder P Tanilon J Are overweight and obese youths more often bullied by their peers&quest; A meta-analysis on the relation between weight status and bullying International Journal of Obesity 2014 10.1038/ijo.2014.117 25002148

[B92] Wu YP Reiter‐Purtill J Zeller MH The Role of Social Support for Promoting Quality of Life Among Persistently Obese Adolescents: Importance of Support in Schools Journal of School Health 2014 84 2 99 105 10.1111/josh.12129 25099424

[B93] Borghese MM Tremblay MS Leduc G Boyer C Bélanger P LeBlanc AG Independent and combined associations of total sedentary time and television viewing time with food intake patterns of 9- to 11-year-old Canadian children Applied Physiology, Nutrition, and Metabolism 2014 39 8 937 943 10.1139/apnm-2013-0551 24892903

